# A procedure for identifying homologous alternative splicing events

**DOI:** 10.1186/1471-2105-8-260

**Published:** 2007-07-19

**Authors:** David Talavera, Adam Hospital, Modesto Orozco, Xavier de la Cruz

**Affiliations:** 1 Molecular Modelling and Bioinformatics Unit, Institut de Recerca Biomèdica (IRB), Parc Científic de Barcelona (PCB), Barcelona, Spain; 2 Protein Structure and Modelling Node, Instituto Nacional de Bioinfomática, Genoma España, Parc Científic de Barcelona, Barcelona, Spain; 3 Departament de Bioquímica i Biologia Molecular, Universitat de Barcelona, Barcelona, Spain; 4 Institució Catalana de Recerca i Estudis Avançats (ICREA), Barcelona, Spain; 5 Computational Biology Program, Barcelona Supercomputing Center, Barcelona, Spain

## Abstract

**Background:**

The study of the functional role of alternative splice isoforms of a gene is a very active area of research in biology. The difficulty of the experimental approach (in particular, in its high-throughput version) leaves ample room for the development of bioinformatics tools that can provide a useful first picture of the problem. Among the possible approaches, one of the simplest is to follow classical protein function annotation protocols and annotate target alternative splice events with the information available from conserved events in other species. However, the application of this protocol requires a procedure capable of recognising such events. Here we present a simple but accurate method developed for this purpose.

**Results:**

We have developed a method for identifying homologous, or equivalent, alternative splicing events, based on the combined use of neural networks and sequence searches. The procedure comprises four steps: (i) BLAST search for homologues of the two isoforms defining the target alternative splicing event; (ii) construction of all possible candidate events; (iii) scoring of the latter with a series of neural networks; and (iv) filtering of the results. When tested in a set of 473 manually annotated pairs of homologous events, our method showed a good performance, with an accuracy of 0.99, a precision of 0.98 and a sensitivity of 0.93. When no candidates were available, the specificity of our method varied between 0.81 and 0.91.

**Conclusion:**

The method described in this article allows the identification of homologous alternative splicing events, with a good success rate, indicating that such method could be used for the development of functional annotation of alternative splice isoforms.

## Background

In recent years, understanding the contribution of alternative splicing (AS) to biological processes has become an active area of research in many fields of biology and biomedicine [1-9]. This has been motivated by the biological relevance of AS, a process shown by a large fraction of human genes (~74%[10]), which results in the diversification of the nature and expression pattern of their corresponding products [2,8]. For instance, it has been found that different alternative splice isoforms of the DSCAM protein are involved in the development of neuronal interconnections by choosing the proper interaction partners [2]. AS is also able to alter the substrate specificity of enzymes by modifying their active site, as previously shown for *Anopheles dirus*'s glutathione S-transferase [3]. In the case of transcription factors, AS plays a regulatory role that has a clear impact on the levels of gene expression [11,12]. The roles of transcription factors isoforms are very broad, and depend on the nature of the sequence changes associated with the AS event [11]: loss of the DNA-binding domain results in isoforms that will act as dominant-negative inhibitors of the corresponding full-length isoforms. In other cases, functional modulation is obtained by small insertions/deletions in the space between DNA-binding domains, etc. All these examples illustrate the importance of understanding the functional role of alternative splice isoforms if the aim is to improve our knowledge of biological processes like development, tissue differentiation, resistance to insecticides, etc.

In addition to its intrinsic biological interest, there is also a major biomedical interest in understanding the functional role of gene isoforms, as deviations from a gene normal AS pattern -either through isoform expression imbalance or presence of aberrant isoforms- are at the origin of many diseases [13,4,14]. Examples cover different cancer types -leukaemia, colon cancer, etc- [15], neurological [1] and immune disorders [16], etc. Availability of functional annotations for AS events is also relevant in applied biomedical research, as these may contribute to the selection of animal models for the above-mentioned diseases given that proper models must show coincidence in the AS patterns of the disease gene with its human ortholog [16]. Lastly, drug design strategies are also starting to include knowledge of the different functional roles of alternative splice isoforms [5], as targeting the wrong isoform may result in unexpected damaging effects [17].

All these facts stress the importance of having functional annotations of AS events and have fuelled bioinformatics research in this field. Indeed, a blossoming of bioinformatics studies has been witnessed in recent years [18-20] which have led to important advances in the enumeration of a gene isoforms [21,19,20,25], in the processing of expression data [10,23,8], in the characterization of the nature of AS changes [26,6,23,28], and in the study of the evolutionary role of AS [29,23,9,33]. However, the functional annotation of AS, or annotation of the biological/biochemical role of the different isoforms expressed by a gene, still remains an open problem [23].

A natural approach to this problem would be to directly determine the functional effects of AS by studying their impact on protein structure. This approach may work in some cases, in particular when sequence changes involve gain/loss of domains of known function [11]. However, when sequence changes are small, or involve substitutions, or domain insertions/deletions in non-annotated parts of the protein, functional inference may be very difficult [23]. For example, in the case of rat Piccolo C_2_A, an apparently innocent insertion results in an isoform with completely unexpected structural modifications [34,23]. Within this context, annotation processes based on data mining the increasingly large amount of experimental information available on AS, may be a good option to obtain information on the functional effects of AS. Implementation of these annotation protocols requires a database of known AS events that can be queried and a method for the identification of homologous [35] or equivalent AS events that will allow the identification of proper candidates in the database.

**Figure 1:**
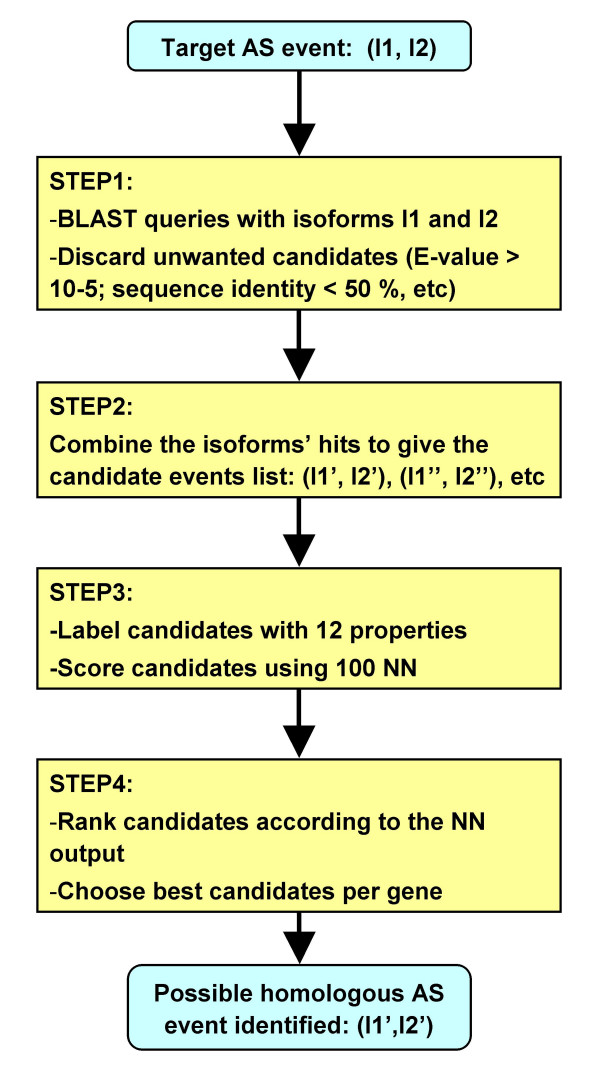
Protocol for the identification of homologous alternative splicing events presented in this work. Our procedure is divided in the four steps shown in the figure: a BLAST search of an isoforms database with each isoform of the target AS event, obtention of a list of candidate events, scoring of the candidates with a set of NN and final filtering of the accepted candidates. A more detailed description is provided in the text of the article. In the article we compare the performance of our method with that of a control method (see **Methods **section) which is an extension of a previously described protocol to find conserved AS events between species [45].

Here we present a protein-level procedure aimed at the identification of homologous AS events, which works irrespective of the nature of the associated sequence change between isoforms, *i.e. *substitution, insertion/deletion, or a mixture of both. The procedure (illustrated in Figure 1) is conceptually similar to standard procedures for protein function annotation. It is based on the utilisation of the two isoforms of a target AS event to query an isoform database. The resulting hits are then combined to give a small set of candidate AS events which are subsequently scored by 100 neural networks (NN). The method has been tested giving an accuracy of 0.99 and a precision and sensibility of 0.98 and 0.93, respectively, confirming that the method constitutes a positive step towards the functional annotation of AS events.

## Results

### Prediction procedure

We define an AS event as a pair of isoforms (I1, I2) from the same gene. Because a gene with AS may express more than two isoforms [7], annotation of the whole AS pattern of the gene would require a repeated application of our method; however we will not address this issue here.

Our goal was to devise a method to find homologous, or equivalent, AS events (I1', I2') of a target event (I1, I2). We followed the definition of homologous AS events from our previous work [35]: two events (I1, I2) and (I1', I2') were called homologues when isoforms I1 and I1' had an equivalent function, with the same being true for isoforms I2 and I2'. Two isoforms were considered to be functionally equivalent when their sequence identity was ≥ 50% [36,37].

Our procedure was designed to work at protein level, where there are only two types of sequence changes associated with AS: substitutions and/or insertions/deletions. It is well known that the size of these changes may vary broadly [29,6,28], and in some cases these changes can be very small [38]. Small changes can be a source of large fluctuations in the parameters we utilise to score the pairs of homologous events (see **Methods **section below), thus negatively affecting the training of the NN. Also, in some cases, small changes may correspond to sequencing/annotation errors [29]. Therefore, to avoid these problems, we eliminated those AS substitution events for which the length of at least one of the substituted sequence stretches was below 10 residues [29]. This minimum-size filter was also extended to the case of insertions/deletions. AS events with insertions/deletions smaller than 10 residues were also excluded. As a result of this filtering, 8.4% of the events were discarded before applying the prediction protocol.

**Figure 2:**
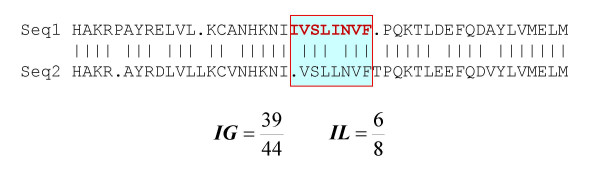
Local and global sequence identities. This figure illustrates how we computed these properties. Global sequence identity is the ratio between the total number of identical residue pairs in the alignment divided by the total number of aligned pairs. Local sequence identity is computed relative to the sequence stretch in the target isoform (upper sequence, red bold characters) modified by alternative splicing, and is equal to the number of identical residue pairs involving residues from this stretch, divided by the size of the stretch (the part of the alignment involved in computing local sequence identity is enclosed within a red box and shaded in light blue).

The final protocol comprised four main steps (Figure 1): a BLAST query of an isoform database with each isoform of the target AS event, obtention of a list of candidate events, scoring of the candidates with a set of NN, and final filtering of the accepted candidates. These steps are described below in more detail:

.- STEP 1. For each isoform of the target event we performed a BLAST [39] query of an isoform database (see **Methods **section below). After excluding hits with E-values above 10^-5^, we kept the best hit for each query according to the BLAST bit score [40]. In case different hits had the same bit score they were all kept. Next, each target isoform was aligned to its recovered candidate (or candidates), using a standard dynamic programming algorithm [41], with gap opening and elongation penalties of -11 and -1, respectively, and the Blosum62 scoring scheme [42]. After alignment, we excluded any hit showing a percentage of sequence identity with the target isoform below 50%. Then, a local sequence identity filter was applied: the percentage of sequence identity at the location of the alternative splicing change had to be ≥ 50% (Figure 2), otherwise the hit was eliminated from the corresponding list. At the end of this step we had two hit lists, one per isoform.

.- STEP 2. We built a set of candidate events by obtaining all possible combinations between the members of each isoform list. Pairs composed of the same isoform were excluded. Pairs composed of isoforms from different genes or different species were also excluded. For example, the search with isoform I1 recovered isoforms I1' and F1', and the search with isoform I2 recovered isoforms I1', I2', I3' and F2'; where I1', I2' and I3' are expressed by gene I, and F1' and F2' by gene F. The final list of candidate homologous events produced at this step was: (I1', I2'), (I1', I3') and (F1', F2'). The pair (I1', I1') was excluded because both isoforms were the same; the pairs (I1', F2'), (F1', I1'), (F1', I2'), (F1', I3') were excluded because the candidate isoforms belonged to different genes.

**Figure 3:**
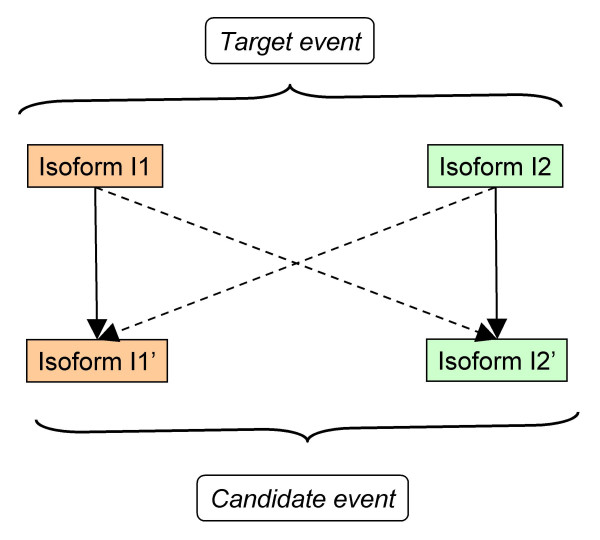
Comparisons between isoforms. For each property used to characterize the candidate events we obtained four values corresponding to four comparisons between the isoforms of the target event and those of the candidate event. The four comparisons are shown in the figure with arrows relating the involved isoforms. Continuous and dashed lines are used for the arrows linking the functionally equivalent (highlighted with the same colour) and non-equivalent isoforms, respectively.

.- STEP 3. For each candidate event obtained in the previous step we computed a set of 12 properties (see **Methods **section below and Figure 3). These 12 properties were the input to 100 NN that produced an output between 0 and 1 (see **Methods **section below). A candidate event was considered to be homologue of the target event when both the ratio (number of NN with output > 0.5)/(number of NN) and the average NN outputs were ≥ 0.5.

.- STEP 4. The candidate events accepted in STEP 3 where then ranked according to the number of NN with output > 0.5, and for each gene the first candidate was kept only. In case two candidates from the same gene ranked equally we further ordered both candidates according to average of their corresponding NN outputs and subsequently took the top candidate.

### Testing method performance

**Figure 4:**
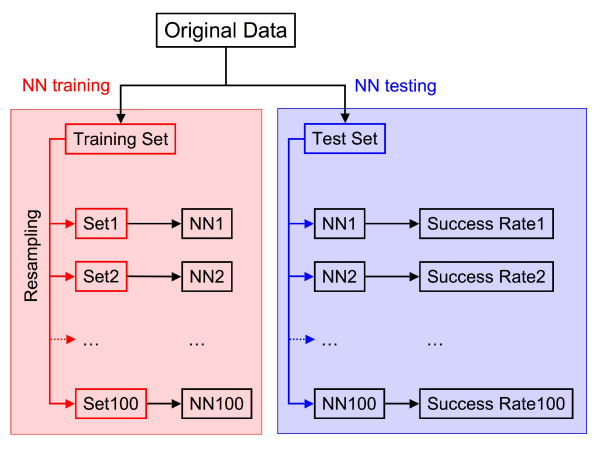
NN training and testing. In the figure we highlight these two processes with a different colour code, red for the training and blue for the testing. We followed a two-fold heterogeneous cross-validation scheme [50] in which the original dataset was split in two (training and test sets). A resampling protocol was applied to correct for class-imbalance effects [51], resulting in 100 training sets with the same proportion of correct and incorrect observations. Each training set was then utilised to train a NN. We applied the latter to the events in the test set and computed the success rate. The success rate given in the article is the average of the success rates for the 200 NN.

We tested our method performance in a set of 473 manually annotated pairs of homologous AS events (see **Methods **section below). The performance figures (see **Methods **section below) given here are an average of the test sets results (Figure 4). In no case we utilised the same data to simultaneously train the NN and assess the performance of the whole method.

The previous test is usually employed to assess the reliability of sequence searches [43,44], giving a good idea of their performance. However, there is at present a clear difference between our problem and the classical problem of querying a sequence database for a protein with the same structure/function. For the latter problem we have nearly achieved a full coverage of the protein structure/function space, and thus there will always be good candidates in the database, irrespective of whether candidates can be recovered or not. In our case, despite great efforts in this direction [8], it is yet unclear how much we know about the alternative splicing pattern of all known genes (independently of the species) [8]. Indeed, on top of the normal limits imposed by the present techniques [8], there may be complex evolutionary phenomena that affect AS conservation among species [31,33,28]. Thus, it is possible for a given AS event not to have any homologues in the isoform database. To assess the ability of our method to recognize this situation and thus produce no false candidates we performed what we called the no-candidate test. This test was implemented in two different fashions: given a target AS event (I1, I2) and its homologous AS event (I1', I2') we eliminated either isoform I1' or isoform I2' from the isoform database or alternatively both isoforms were eliminated. In neither of these two versions of the test was it possible to recover the correct hit, and therefore any candidate recovered would have corresponded to a method error. The test was applied using the set of 473 correct cases.

### Performance results

We first show the results for the NN, which is at the core of our approach, and then those for the whole method. The latter are shown together with those corresponding to the control method (see **Methods **section).

Table 1 shows the results for NN performance. These results indicate that the variables chosen for recognising homologous AS events work well, resulting in an accuracy of 0.89 and a precision and specificity of 0.46 and 0.94, respectively. To assess the ability of NN to detect difficult cases of incorrect pairings, we replaced the original incorrect pairs of AS events in the test set by a collection of 86 hard cases (see **Methods **section). Please, note that no retraining of NN was done using the latter data. We observe (Table 1) that, despite the challenging nature of the incorrect pairings, NN retain a good performance, with an accuracy of 0.82 and a precision and sensitivity of 0.83 and 0.94, respectively. We can see that for this second test the precision was better than that of the first test, 0.83 vs. 0.46, whilst this trend was reversed for specificity, 0.47 vs. 0.88. This is due to the fact that the population of incorrect homologous AS events was smaller in the second case. Overall, these data show the ability of NN to discriminate between correct and incorrect pairs of AS events.

**Table 1:** NN performance. The results given in this table illustrate the ability of the trained NN, which are at the core of our method, to distinguish between correct from incorrect pairs of AS homologous events. The results in the second column correspond to the cross-validated performance of NN when tested using 473 and 4746 correct and incorrect pairs of homologous AS events, respectively. The results in the third column were obtained after replacing the 4746 incorrect pairs with a small set of 86 of hard-to-identify incorrect pairs. In both cases NN was shown to have a good recognition ability.

	True positives + Built negative cases	True positives + Hard negative cases
Accuracy	0.89 ± 0.01	0.82 ± 0.02
Precision	0.46 ± 0.06	0.83 ± 0.01
Sensitivity	0.94 ± 0.02	0.94 ± 0.02
Specificity	0.88 ± 0.01	0.47 ± 0.05

If we now consider the whole method we can see (Table 2) that the performance is also good, with an accuracy of 0.99 and a precision and a sensitivity of 0.98 and 0.93, respectively. The 0.98 precision shown by the method is substantially higher than that of NN, 0.46. This is caused by (i) a smaller number of incorrect pairs of AS events reaching NN due to the filters applied to the results of the BLAST searches in STEP 1, and (ii) to the filter applied in STEP 4, where only the best candidate event was chosen. If we now compare the performance of our method with that of the control method (Table 2), we see that while the former has higher accuracy, 0.99 (our method) vs. 0.96 (control method), it has a slightly lower sensitivity 0.93 (our method) vs. 0.97 (control method). This indicates that slightly less candidates were recovered with our method due to the more stringent nature of our prediction protocol, which in some cases may lead to the rejection of good candidates. However, this also results in a clearly better ability of our method to reject false positives, as shown by the precision values 0.98 and 0.73, for our method and the control method, respectively.

**Table 2:** Method performance. The results given in this table show the ability of the whole method to identify AS homologous events. The results in the second column correspond to our method, while those in the third column correspond to a simple control method in which no NN was utilised (see Methods section). Both methods have a high accuracy, but our procedure displays a better precision, related to its ability to discard wrong candidates.

	OUR METHOD	CONTROL METHOD
Accuracy	0.99 ± 0.00	0.96 ± 0.04
Precision	0.98 ± 0.01	0.73 ± 0.31
Sensitivity	0.93 ± 0.02	0.97 ± 0.01
Specificity	1.00 ± 0.00	0.96 ± 0.05

Overall, these results indicate that in general given a target AS event our method will be usually successful at finding a homologous candidate event (Table 2) for as long as there is at least one candidate in the isoform database. To assess the performance of our method when no candidate was available in the database, we performed a no-candidate test (see above), in which all good candidates of the target event were removed from the isoform database. We see (Table 3) that for both versions of the test, our method always showed a better specificity than the control method, confirming its better ability to discard false positives.

**Table 3:** The no-candidate test. This test was devised to measure the ability of our method to identify and discard incorrect pairs of AS events. Specificity was used as performance measure (equation 4). We observed that in both versions of the test the specificity of our method was better than that of the control method (see Methods section), indicating a better ability of the former to discard false positives.

	OUR METHOD	CONTROL METHOD
Lack of one isoform	0.81 ± 0.04	0.71 ± 0.04
Lack of both isoforms	0.91 ± 0.02	0.88 ± 0.05

### Error analysis

**Figure 5:**
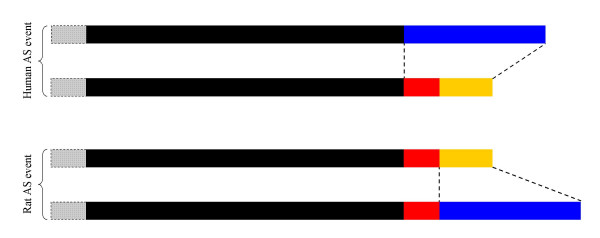
Example of false positive. The figure shows two AS events constituted by one long and one short isoform each. One is from human ATP2B3, the other from rat Atp2b3. These two events are not homologous, although our method predicts them as such. This is probably because the human long/mouse long and human short/mouse short isoform pairings have much better values of the scoring parameters than the human long/mouse short and human short/mouse long pairings. Dotted lines between isoforms indicate sequence stretches substituted between isoforms in each species. Black: protein sequence adjacent to the AS sequence change. Blue: substituted sequence stretch from the long isoform. Red: sequence stretch from the short isoform substituted only in human. Yellow: sequence stretch from the short isoform substituted in both species. Light grey: remaining of the protein, where a second and identical AS sequence change takes place in both species.

In general the success of our method, and in particular its ability to identify good hits, will depend on NN performance. If the pairings between equivalent isoforms are sufficiently different from those between incorrect isoforms, NN will easily identify good candidates. However, when this is not the case these candidates will go unnoticed resulting in false negatives. For example, in the case of RAB6A (Ras-related protein Rab-6A) there is an AS event, corresponding to a substitution, which is homologous between human (SwissProt code: RAB6A_HUMAN) and mouse (SwissProt code: RAB6A_MOUSE). In this case global and local sequence identities for the correct pairings -global: 99% and 99%, local: 100% and 100%- are almost as good as those for the incorrect pairings -global: 97% and 97%, local: 96% and 96%. As a result, the NN is unable to decide whether there is a preferred pairing, and therefore discards the mouse event as a homologue of the human event. The contrary occurs when substantial differences appear between alternative isoform pairings; in this case even though none of the pairings is correct, the NN accepts wrong candidates as correct. This is what happens for ATP2B3 (Plasma membrane calcium-transporting ATPase 3), one of the 86 incorrect pairs of homologous AS (see **Methods **section). The AS events in human and mouse are not homologous (Figure 5), even though they are predicted as such by our method. This is because one of the possible isoform pairings gives better scoring parameters than the other, leading the NN to a positive prediction.

We tested our prediction protocol to determine whether we could find any particular bias in the AS events correctly identified. We considered four possible sources of bias: similarity between isoforms (percentage of sequence identity between equivalent isoforms in homologous AS events), nature of the AS event (events with insertions/deletions, substitutions or both), size of the insertion/deletion (subdomain size: <30 residues/domain size: ≥ 30 residues) and sequence location of the AS change. We found (Table 4) a dependence on the similarity between isoforms, with precisions ranging from 0.83 to 0.96, for average similarities between 60% and 90%, respectively. The differences were smaller when we considered the nature of the event, with precisions of 0.91, 0.96 and 0.99, for insertions/deletions, substitutions and complex events (mixtures of insertions/deletions and substitutions), respectively. The better performance observed for complex events is due to the fact that equivalence between isoforms requires fulfilment of more conditions. In the case of insertions/deletions (the most frequent sequence change associated with AS) we found that the performance of the method was essentially independent of size. When considering sequence location of AS change we only observed very little performance differences. Overall, despite the existence of some small trends, the performance of the method remained substantially high.

**Table 4:** Error analysis. Four sources of bias were considered: average identity between equivalent isoforms, AS mechanism, size of the insertion/deletion, and sequence change location, which can be external (AS involves at least one sequence terminus), internal (no sequence terminus is affected by AS) and external+internal (AS changes happen at both external and internal locations). Whilst a certain trend may be observed for the first case, the performance of the method is nonetheless high. Only small performance changes are observed for AS mechanism, size of the insertion/deletion andsequence change location.

**Average identity between equivalent isoforms (sample size)**	**Accuracy**	**Precision**	**Sensitivity**
90% (N = 305)	0.99 ± 0.00	0.96 ± 0.02	0.95 ± 0.01
80% (N = 89)	0.98 ± 0.02	0.89 ± 0.06	0.89 ± 0.06
70% (N = 25)	0.93 ± 0.04	0.83 ± 0.05	0.83 ± 0.05
60% (N = 13)	0.95 ± 0.06	0.83 ± 0.24	0.83 ± 0.24

**AS mechanism (sample size)**	**Accuracy**	**Precision**	**Sensitivity**

Insertions/deletions (N = 248)	0.98 ± 0.01	0.91 ± 0.03	0.90 ± 0.02
Substitutions (N = 147)	0.99 ± 0.01	0.96 ± 0.04	0.95 ± 0.03
Complexes (N = 78)	1.00 ± 0.00	0.99 ± 0.02	0.99 ± 0.02

**Insertion/deletion size (sample size)**	**Accuracy**	**Precision**	**Sensitivity**

Small (N = 145)	0.98 ± 0.00	0.90 ± 0.01	0.90 ± 0.00
Big (N = 103)	0.98 ± 0.01	0.91 ± 0.05	0.91 ± 0.05

**AS region position (sample size)**	**Accuracy**	**Precision**	**Sensitivity**

External (N = 153)	0.99 ± 0.01	0.95 ± 0.06	0.95 ± 0.06
Internal (N = 235)	0.99 ± 0.01	0.91 ± 0.02	0.90 ± 0.00
External+Internal (N = 85)	1.00 ± 0.01	0.99 ± 0.02	0.99 ± 0.02

Finally, it must be pointed out that genes with a larger number of isoforms are more prone to give false positive hits than genes with only a few isoforms, independently of the existence of any kind of bias.

## Discussion and conclusion

We have developed a method for the identification of homologous, or equivalent, AS events based on the combined use of NN and sequence searches. The method works at protein level, where AS changes are either insertions/deletions and/or substitutions. Its performance is reasonably good when tested under different conditions (presence or absence of the homologue AS event in the isoform database), regardless of whether we consider its accuracy or ability to discard false positive hits. We have also compared the performance of our method with that of a simple control method in which the use of NN was eliminated. This control method is an adaptation to the protein level of a previously described strategy for finding conserved AS events [45], which we have extended to cover the full range of AS events. We observe that while the accuracy of both methods is comparable, our approach has a better ability to discard false positives, due to the presence of the neural network. These results indicate that our method constitutes a positive step towards the development of protocols for the functional annotation of AS events using information from public databases.

## Methods

### The isoforms database

This database included the sequence of all alternative splice isoforms listed in version 43 of SwissProt [46]. However, the method is independent from the origin of AS data, and these can come from other databases like ENSEMBL [47], ASAP [18], etc.

### Alternative splicing sequence changes

As mentioned above, our procedure was devised to work at protein level, where sequence changes associated with AS are of two types: substitutions and/or insertions/deletions. However, because AS takes place at pre-mRNA level, Table 5 includes, as a reference for the reader, the correspondence between pre-mRNA and protein sequence changes, for the most frequent AS events.

**Table 5:** Correspondence between protein and mRNA level sequence changes. Pre-mRNAs can be alternatively spliced in several ways [53] [54]. The corresponding sequence changes map only to two types of sequence changes at protein level: substitutions and/or insertions/deletions. In this table we show the ten most frequent types of pre-mRNA AS (first column) and the corresponding protein sequence changes (second and third columns). The former where obtained from the work of Nagasaki and colleagues [54], after grouping some of their types, and are described using a notation similar to that used by Zheng and coworkers [53].

AS type	Insertion/deletion	Substitution
Alternative transcriptional initiation	No	Yes
Exon skipping	If exon size is multiple of 3	If frameshift changes
Alternate polyadenilation site	No	Yes
Intron retention	If intron size is multiple of 3	If frameshift changes
Alternative acceptor	If frameshift is preserved	If frameshift changes
Alternative donor	If frameshift is preserved	If frameshift changes
Multiple exon skipping	If global exon size is multiple of 3	If frameshift changes
Mutually exclusive exons	No	Yes
Complex Alternative donor/Exon skipping or Alternative acceptor/Exon skipping	If frameshift is preserved	If frameshift changes
Complex Multiple exon skipping/Alternate polyadenilation site	No	Yes

Application of our method required knowing exactly the sequence change associated with the target AS event, *i.e. *the number, size and location of insertions/deletions as well as the number, size and location of substitutions. The information on these changes was given relative to one isoform from the pair constituting the AS event, following SwissProt [46]. That is, if our target event was constituted by the isoform pair (I1, I2) and I1 was taken as reference, the sequence changes between both isoforms were defined relative to the sequence of I1. For example, if the AS event involved a sequence substitution, we located the substituted sequence stretch in I1, defining its size and the replacing stretch.

### The properties

Candidate events were characterized with a set of properties (STEP 3 of the method) that were utilized by NN to predict whether they could be homologues of the target event. Three properties were utilized to this end: global percentage of sequence identity, local percentage of sequence identity and size ratio. Four values were obtained for each property corresponding to the following isoform comparisons (Figure 3): I1-I1', I1-I2', I2-I1' and I2-I2' (where I1 and I2 were the isoforms defining the target event, and I1' and I2' those defining the candidate event). Thus, a total of 12 values were associated with each candidate event. We describe below the properties utilized and how these were computed.

#### Global percentage of sequence identity

This is the standard percentage of sequence identity, obtained after sequence alignment of the involved isoforms. The sequence alignment was produced with the Needleman & Wunsch algorithm [41]. The percentage of sequence identity was computed as: 100.(number of identical residue pairs)/(total number of aligned residue pairs).

#### Local percentage of sequence identity

To compute this parameter we used the information on sequence changes among isoforms in the target event (see above). This local sequence identity was computed as (Figure 2): 100.(number of identical residue pairs involving residues from the "modified sequence stretch")/(size of the "modified sequence stretch" in the corresponding isoform of the target AS). The "modified sequence stretch" was that part of the target isoform sequence affected by alternative splicing (Figure 2). If the AS event was a substitution, there were two affected sequence stretches, one per isoform, that resulted in four local percentages of sequence identity, one for each of the above-mentioned comparisons: I1-I1', I1-I2', I2-I1' and I2-I2'. If the AS event was an insertion/deletion, only two local sequence identities were computed using the affected fragment: for example, if the target AS event involved a deletion in isoform I1, then local sequence identities were only computed for this stretch. The local sequence identities involving isoform I2 were arbitrarily set to 0. This is of course an arbitrary decision, and a more refined method can probably be obtained by considering substitutions and insertions/deletions separately.

For complex events involving more than one sequence change, e.g. two substitutions, we averaged the four values of the local sequence identities over all the changes, to give again 4 values for this parameter.

#### Size ratios

Finally, we computed the size ratios between isoforms as: (number of residues of the candidate isoform)/(number of residues of the target isoform), for the comparisons: I1-I1', I1-I2', I2-I1' and I2-I2'.

### The neural network

All the candidates recovered at the end of STEP 2 were scored using their properties vectors as input to a set of 100 feed-forward NN. Each of these NN produced an output that is a number between 0 and 1, with values close to 0 or 1 corresponding to bad or good candidates, respectively.

Each of the 100 NN had the same structure and comprised a single hidden layer of two units, resulting in a total of 29 weights. These weights were computed presenting the NN with a number of inputs together with their associated target outputs [48,49]. The final weights were the result of 500 optimisation steps using scaled conjugate gradients.

### Training of the neural networks

Each NN was trained to discriminate between homologous and non-homologous candidates. To this end, it was presented with a set of inputs of both kinds. In the next two sections we describe how we obtained this dataset and the cross-validation protocol followed for the training of the NN.

#### 1. The input data set

The pairs of homologous events were obtained manually, following a previously described protocol [35] that combined visual inspection of AS events between different species with information from the literature, when possible. For completion, we describe this procedure again. First, we recovered a list of AS events querying the SwissProt database [46] with the keyword VARSPLIC (note that in recent versions of the database this keyword has been replaced by keyword VAR_SEQ). Then, we grouped the recovered AS events according to the gene affected. We subsequently explored these groups to find pairs of homologous events, by looking for AS events showing comparable sequence changes both in nature (e.g. insertions/deletions or substitutions) as well as in location. In addition, we also decided that global and local sequence identities between equated isoforms should be ≥ 50%, to avoid recognition problems in the sequence twilight zone. Finally, when possible, we utilized functional evidence from the literature regarding, for example, differential expression or biological activity of the different isoforms of a gene. At the end of this procedure we recovered a total of 473 pairs of homologous events, corresponding to 321 genes from 17 species.

The pairs of non-homologous events were built to reflect the most frequently expected incorrect isoform pairings, applying two different procedures to the 473 pairs of homologous events. In the first procedure, for each pair of homologous events, we produced a pair of non-homologous events by switching the isoforms in the second event. That is, if we had a pair of homologous events (I1, I2) and (I1', I2') we replaced the latter with (I2', I1'). In the second procedure pairs of non-homologous events were produced by modifying the isoforms of the second AS event. For example, we started with the correct pairing of events (I1, I2) and (I1', I2'), and replaced the latter with (I1', I3'). This procedure required that at least one of the genes had more than two isoforms. The final number of pairs of non-homologous events was 4746.

The total number of events in the input dataset was 473 correct assignments and 4746 incorrect assignments.

#### 2. The cross-validation procedure

We followed a two-fold cross-validation scheme (Figure 4) in which the previous dataset was split in two, imposing that all data from one gene were in the same set, following a stringent heterogeneous cross-validation scheme [50]. In standard cross-validation one of the resulting sets is used to train the NN and the other is used to test its performance, then the procedure is repeated again after switching sets. However, in our case the split sets reflected the imbalance between correct, 473 cases, and incorrect pairings between AS events, 4746 cases. Because imbalanced training sets may result in biased predictors [51] we applied an oversampling procedure to generate a new, well-balanced, training dataset [51]. This was done by keeping all the cases from the most frequent class (incorrect pairings) and by increasing the less frequent class (correct pairings) until reaching a 1:1 ratio. The latter was done by randomly sampling the set of correct cases from the original training set. For example, if the original training set had 100 and 1000 correct and incorrect pairings, respectively, we built a new training set with a total of 2000 pairs by increasing the number of correct cases, randomly replicating the original 100 elements, until a total of 1000 was reached. Then, we trained NN with the resulting new set. It is important to note that the resampling procedure was only applied to the training set. This procedure was repeated 100 times for each training set, resulting in 100 trained, different NNs. The performance figures shown here are an average of the 200 results for the test sets (100 for each of the two test sets).

An additional test was carried out to assess the ability of NN to discard false positives resembling true hits. To this end, the performance of each NN was assessed in a new test set in which the correct pairs of AS events were maintained, but the incorrect pairs were replaced with those from a small set of 86 incorrect pairs of homologous AS events. The latter correspond to pairs of events that were discarded when building the set of 473 correct cases but which are similar to these, and are therefore particularly challenging.

The performance figures shown are an average over the results of the test sets.

### A control method

We utilised an alternative method to control the improvement introduced by the use of NN. In this alternative method only the BLAST [39] search with both isoforms was done, and the candidate event for a given gene and species was constituted by the best hit found for each target isoform (after excluding those hits with E-values above 10^-5^) according to the BLAST bit score [40]. If more than one hit had the same bit score, they were all kept. In summary, the control method is essentially a restriction to STEPS 1 and 2 of our identification method (see **Results **section).

This method bears some resemblance to and can be considered an extension of a protocol recently described by Pan and co-workers [45], designed to find conserved AS events between human and other species. However, their method works at DNA level and focuses on exon-skipping events [45], whilst our control method works at protein level and is not limited to any kind of AS event, to allow a proper comparison with our NN-based prediction protocol.

### Performance measures

We utilised the following parameters: accuracy (equation 1), precision (equation 2), sensitivity (equation 3), and specificity (equation 4). These parameters are routinely used to assess the performance of pattern recognition methods when applied to biological problems, in particular when one class (in our case the true homologous AS event) is more relevant than the others [52]. The symbols *tp*, *tn*, *fp *and *fn *used below correspond to the number of true positives, true negatives, false positives and false negatives, respectively.

accuracy=tp+tntp+tn+fp+fn
 MathType@MTEF@5@5@+=feaafiart1ev1aaatCvAUfKttLearuWrP9MDH5MBPbIqV92AaeXatLxBI9gBaebbnrfifHhDYfgasaacH8akY=wiFfYdH8Gipec8Eeeu0xXdbba9frFj0=OqFfea0dXdd9vqai=hGuQ8kuc9pgc9s8qqaq=dirpe0xb9q8qiLsFr0=vr0=vr0dc8meaabaqaciaacaGaaeqabaqabeGadaaakeaacqWGHbqycqWGJbWycqWGJbWycqWG1bqDcqWGYbGCcqWGHbqycqWGJbWycqWG5bqEcqGH9aqpdaWcaaqaaiabdsha0jabdchaWjabgUcaRiabdsha0jabd6gaUbqaaiabdsha0jabdchaWjabgUcaRiabdsha0jabd6gaUjabgUcaRiabdAgaMjabdchaWjabgUcaRiabdAgaMjabd6gaUbaaaaa@4D00@

precision=tptp+fp
 MathType@MTEF@5@5@+=feaafiart1ev1aaatCvAUfKttLearuWrP9MDH5MBPbIqV92AaeXatLxBI9gBaebbnrfifHhDYfgasaacH8akY=wiFfYdH8Gipec8Eeeu0xXdbba9frFj0=OqFfea0dXdd9vqai=hGuQ8kuc9pgc9s8qqaq=dirpe0xb9q8qiLsFr0=vr0=vr0dc8meaabaqaciaacaGaaeqabaqabeGadaaakeaacqWGWbaCcqWGYbGCcqWGLbqzcqWGJbWycqWGPbqAcqWGZbWCcqWGPbqAcqWGVbWBcqWGUbGBcqGH9aqpdaWcaaqaaiabdsha0jabdchaWbqaaiabdsha0jabdchaWjabgUcaRiabdAgaMjabdchaWbaaaaa@437F@

sensitivity=tptp+fn
 MathType@MTEF@5@5@+=feaafiart1ev1aaatCvAUfKttLearuWrP9MDH5MBPbIqV92AaeXatLxBI9gBaebbnrfifHhDYfgasaacH8akY=wiFfYdH8Gipec8Eeeu0xXdbba9frFj0=OqFfea0dXdd9vqai=hGuQ8kuc9pgc9s8qqaq=dirpe0xb9q8qiLsFr0=vr0=vr0dc8meaabaqaciaacaGaaeqabaqabeGadaaakeaacqWGZbWCcqWGLbqzcqWGUbGBcqWGZbWCcqWGPbqAcqWG0baDcqWGPbqAcqWG2bGDcqWGPbqAcqWG0baDcqWG5bqEcqGH9aqpdaWcaaqaaiabdsha0jabdchaWbqaaiabdsha0jabdchaWjabgUcaRiabdAgaMjabd6gaUbaaaaa@468B@

specificity=tntn+fp
 MathType@MTEF@5@5@+=feaafiart1ev1aaatCvAUfKttLearuWrP9MDH5MBPbIqV92AaeXatLxBI9gBaebbnrfifHhDYfgasaacH8akY=wiFfYdH8Gipec8Eeeu0xXdbba9frFj0=OqFfea0dXdd9vqai=hGuQ8kuc9pgc9s8qqaq=dirpe0xb9q8qiLsFr0=vr0=vr0dc8meaabaqaciaacaGaaeqabaqabeGadaaakeaacqWGZbWCcqWGWbaCcqWGLbqzcqWGJbWycqWGPbqAcqWGMbGzcqWGPbqAcqWGJbWycqWGPbqAcqWG0baDcqWG5bqEcqGH9aqpdaWcaaqaaiabdsha0jabd6gaUbqaaiabdsha0jabd6gaUjabgUcaRiabdAgaMjabdchaWbaaaaa@4629@

## Abbreviations

AS: alternative splicing; NN: neural network.

## Authors' contributions

DT obtained the set of manually curated data and developed and tested the method. AH prepared a robust version of the algorithm and participated in testing the method. MO contributed in the design and testing of the method and in the drafting of the manuscript. XD conceived the prediction method, designed most of the testing and wrote the article. All authors read and approved the final manuscript.
